# Tuberculous Joint Lesions

**Published:** 1900-09-22

**Authors:** 


### TUBERCULOUS JOINT LESIONS.

In addressing the Canadian Medical Association at Ottawa on the Clinical Aspects of Tuberculous Lesions, Mr. Edmund Owen made some interesting observations regarding tuberculous joint disease, which are especially worthy of note at the present day when so strong a tendency is shown by many surgeons to regard such tuberculous diseases as always requiring operative treatment. He drew attention to the frequency with which tuberculous disease of the spine, which has gone so far as to produce angular curvature, severe and destructive as it may have been, gets well in the end. Everyone, he says, knows some example of this, or if he does not know him personally he has seen him in the street. "He is rather a short man, with peculiarly high square shoulders, and with a boss between them. And not only has he long since outgrown his tuberculous lesion without any operative assistance whatever, but could we see him in his own home we might not improbably find him—and I say it with some regret—surrounded by a number of apparently healthy sons and daughters." Such a case then is of considerable clinical importance, showing, as it does, that a man with an undoubted tuberculous lesion of the first magnitude can completely recover without having undergone any operative procedure. Another instance of the favourable course which severe tuberculous disease may run in the absence of active surgical interference is seen in the case of old-standing hip-joint disease, the boy actually "growing out of his trouble." When he becomes a young man he may walk lame, and may be precluded from taking an active part in athletics, yet he may be vigorous, and it may be evident that he has quite triumphed over his disease. Turning now to the nature of the tuberculous joint disease and the character of the operation which may be required in certain cases, Mr. Owen said (*a*) that chronic inflammation of a joint in a child or young person was always tuberculous—except in those very rare cases in which it was due to hereditary syphilis or osteo-arthritis; (*b*) that tuberculous inflammation might completely destroy a joint, and then leave it solidly and soundly synostosed without the surrounding tissues of the skin having been implicated; (*c*) that if tuberculous granulation tissue broke down into a fluid, that fluid was not pus, and the collection was not, properly speaking, an abscess—unless by bad fortune or by worse surgery it had become infected by septic micro organisms; and (*d*) that such a fluid collection was not to be treated as an abscess—by incision and drainage—but was to be opened and emptied, and scraped and cleansed of its unhealthy lining of granulation tissue. The wound in the skin was to be completely closed by sutures, firm pressure was to be applied, and the part was to be kept absolutely at rest. Lastly, he said that he had failed to discover that iodoform was of any peculiar value in the treatment of tuberculous lesions.

